# Genomic Landscape Survey Identifies *SRSF1* as a Key Oncodriver in Small Cell Lung Cancer

**DOI:** 10.1371/journal.pgen.1005895

**Published:** 2016-04-19

**Authors:** Liyan Jiang, Jiaqi Huang, Brandon W. Higgs, Zhibin Hu, Zhan Xiao, Xin Yao, Sarah Conley, Haihong Zhong, Zheng Liu, Philip Brohawn, Dong Shen, Song Wu, Xiaoxiao Ge, Yue Jiang, Yizhuo Zhao, Yuqing Lou, Chris Morehouse, Wei Zhu, Yinong Sebastian, Meggan Czapiga, Vaheh Oganesyan, Haihua Fu, Yanjie Niu, Wei Zhang, Katie Streicher, David Tice, Heng Zhao, Meng Zhu, Lin Xu, Ronald Herbst, Xinying Su, Yi Gu, Shyoung Li, Lihua Huang, Jianren Gu, Baohui Han, Bahija Jallal, Hongbing Shen, Yihong Yao

**Affiliations:** 1 Department of Pulmonary, Shanghai Chest Hospital, Shanghai Jiao Tong University, Shanghai, China; 2 Medimmune, Gaithersburg, Maryland, United States of America; 3 Department of Epidemiology and Biostatistics, Collaborative Innovation Center of Cancer Medicine, Jiangsu Key Lab of Cancer Biomarkers, Prevention and Treatment, School of Public Health, Nanjing Medical University, Nanjing, China; 4 Asia & Emerging Markets iMed, AstraZeneca R&D, Shanghai, China; 5 Beijing Genomics Institute, Shenzhen GuangDong, China; 6 Shanghai Cancer Institute, Renji Hospital, Shanghai Jiaotong University School of Medicine, Shanghai, China; Dana Farber Cancer Institute, UNITED STATES

## Abstract

Small cell lung cancer (SCLC) is an aggressive disease with poor survival. A few sequencing studies performed on limited number of samples have revealed potential disease-driving genes in SCLC, however, much still remains unknown, particularly in the Asian patient population. Here we conducted whole exome sequencing (WES) and transcriptomic sequencing of primary tumors from 99 Chinese SCLC patients. Dysregulation of tumor suppressor genes *TP53* and *RB1* was observed in 82% and 62% of SCLC patients, respectively, and more than half of the SCLC patients (62%) harbored *TP53* and *RB1* mutation and/or copy number loss. Additionally, Serine/Arginine Splicing Factor 1 (*SRSF1*) DNA copy number gain and mRNA over-expression was strongly associated with poor survival using both discovery and validation patient cohorts. Functional studies *in vitro* and *in vivo* demonstrate that SRSF1 is important for tumorigenicity of SCLC and may play a key role in DNA repair and chemo-sensitivity. These results strongly support SRSF1 as a prognostic biomarker in SCLC and provide a rationale for personalized therapy in SCLC.

## Author Summary

SCLC patients are initially highly chemo-sensitive with response rates of greater than 80% in both limited and extensive diseases, but suffer uniform disease recurrence or progression in a very short period of time. In the absence of well-defined genomic biomarkers and insights into the resistance mechanism, many targeted treatments have yielded negative results in the last decade Using integrated next generation sequencing (NGS) technology in combination with a high quality surgical sample set with comprehensive clinical annotation, our study not only identified novel recurrent genetic alterations in genes such as CDH10 and DNA repair pathways which may influence outcomes in SCLC patients, but also discovered the expression of SRSF1, an RNA-splicing factor which can both regulate key oncogenic and survival pathways such as BCL2, and play a critical role in patient survival.

## Introduction

Small cell lung cancer (SCLC) represents 13% of all newly diagnosed cases of lung cancer worldwide with more than 180,000 cases per year [1]. It is an aggressive neuroendocrine malignancy with a unique natural history of a short doubling time, high growth fraction, and early development of widespread metastases [2]. Most patients are very sensitive to thoracic radiotherapy and platinum drugs such as cisplatin and carboplatin, but suffer disease recurrence or progression in a very short period of time following initial treatment [1]. Currently, for recurrent or progressive SCLC, the only drug approved in the United States and Europe is topotecan, a topoisomerase 1 (*Top1*) inhibitor which provides some benefit, though the five year survival rate of SCLC has remained unchanged at~5% for the last four decades [2].

To improve patient outcomes in SCLC, it is critical to understand the key genetic alterations that contribute to the specific disease phenotypes and their utility for potential therapeutic targets. However, systematic genetics and genomics analyses of large cohorts of SCLC patients remains a challenge, primarily because SCLC usually presents as extensive disease upon diagnosis and hence is rarely treated surgically, thus causing a lack of suitable tumor specimens for comprehensive analysis. To date, these types of extensive genome-wide molecular analyses have been performed on relatively small patient cohorts, which provide utility restricted to the disease population sampled [3, 4, 5]. Within these studies, among genes recurrently affected by genomic alterations in SCLC, *TP53*, *RB1*, as well as the amplification of *MYC* family members and *SOX2* have been identified. However, the molecular factors related to chemo-sensitivity or resistance remain unknown. Additionally, clinical outcome such as survival in relation to genetic alterations remains unreported, particularly in the SCLC Chinese patient population.

Here, we conducted the first comprehensive genetic landscape survey of Chinese SCLC patients with whole exome sequencing (WES) and transcriptomic sequencing of primary tumors from 99 SCLC patients with detailed clinical history and survival data. Our study not only identified novel recurrent genetic alterations such as *CDH10* and DNA repair pathways which may influence outcomes in SCLC patients, but also revealed *SRSF1*, an RNA-splicing factor which can form complexes with TP53 and Top1, and plays a critical role in SCLC patient survival.

## Results

### Recurrent mutations in SCLC Chinese patients

WES of 25 normal [normal adjacent tissue (NAT) or blood] and matched tumor pairs, and 74 tumors only (no normal tissue) from Chinese SCLC patients revealed 32,566 somatic non-silent single nucleotide variants (SNVs) or insertion/deletions (indels), an average of 329 per patient and non-silent/silent ratio of 2.11. The patient summary is described in **Table 1**and **S1 Table**. The most frequent transition and transversion changes were G>A and G>T, respectively, consistent with a previous report in SCLC [2]. Genes harboring the most recurrent somatic SNVs or indels were *TP53* (82%), *RB1* (47%), *CSMD3* (47%), *NOTCH1* (18%) and *NOTCH3* (15%) (**S2 Table**). *TP53* and *RB1* have been reported previously as the most recurrent genes harboring nonsilent somatic SNVs in SCLC [2,3,4]. Oncogenic gain-of-function mutations in *NOTCH1* commonly occur in human T-cell acute lymphocytic leukemia (T-ALL) and B-cell chronic lymphocytic leukemia [6,7,8]. Loss-of-function mutations in Notch receptors have been recently reported to likely play a tumor suppressor role in lung squamous cell carcinoma and SCLC patients [9, 10]. Additionally, the concordance between the top 100 genes harboring the most recurrent nonsilent somatic SNVs or indels in this study and a recent WES study of Asian SCLC patients (Japanese; n = 51) was 62% (**S2 Table**), with strong consistency of recurrence prevalence in *TP53* (82% vs. 80%), *RB1* (47% vs. 39%), and *CSMD3* (47% vs. 37%), among other genes, between the two studies [5].

**Table 1:** Summary of clinical features of SCLC patients.

Patients	n = 99 (Chinese)
	No. (%)
**Gender**	
Male	86(87%)
Female	13(13%)
**Age (years)**	
Mean	57.92
Median	57
Range	36–78
**Outcome**	
Follow-up (months)	1–66.2
Median follow-up(months)	21.3
Death	43(43%)
Alive	52(53%)
Lost to Follow-Up	3(3%)
**Stage**	
I	18(18%)
II	15(15%)
III	62(63%)
IV	4(4%)
**Cigarette Smoking**	
Smoker	75(76%)
Non-smoker	24(24%)
**Precure Neochemotherapy**	
Treated	8(9%)
Naïve	91(91%)
**Specimens**	
Tumor sample with matched normal	25(25%)
Tumor sample only	74(75%)
**Sequencing summary**	
Exon seq	99(100)
RNA seq	50(50%)

To further narrow down the most disease-relevant mutated genes, we first generated a list of genes harboring the most recurrent and significant nonsilent somatic mutations (identified with two independent algorithms). Then this list was intersected with two independent lists of significantly mutated genes in SCLC generated by both Peifer et al [4] and Umemura et al [5] studies. Aside from *TP53* and *RB1*, neural cell transmembrane genes *TMEM132D*, *NCAM2*, and *CDH10* were shared in all three independent studies (**S3 Table**).The mutation rates of *TMEM132D*, *NCAM2*, *CDH10* in our Chinese patient cohort were 14%, 13% and 12%, respectively.

**Fig 1:**
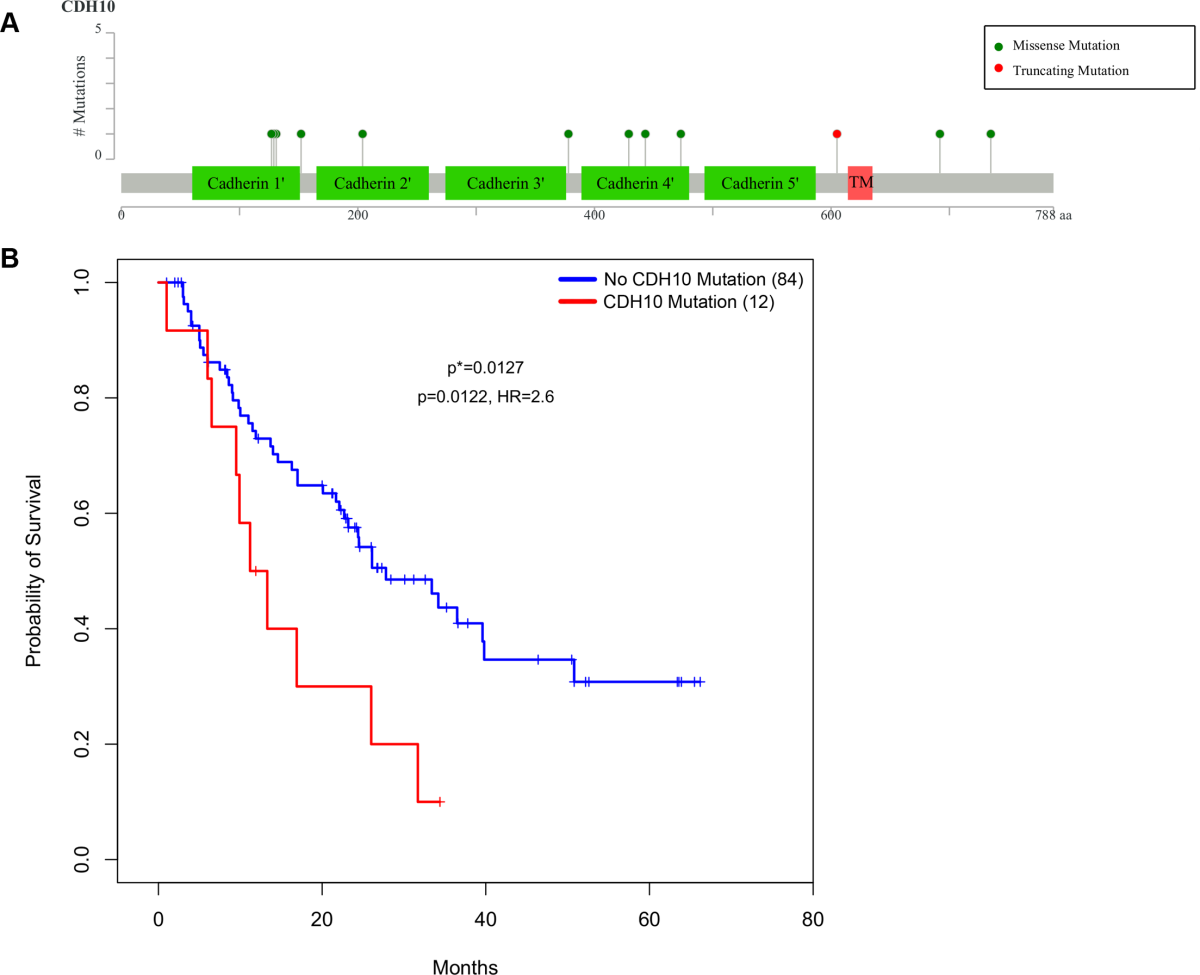
Mutations in *CDH10* associate with poor survival in Chinese SCLC patients. a) Schematic representation of amino acid consequences from mutations identified in SCLC patients in human CDH10 protein b) Kaplan-Meier (KM) curves comparing survival between patients harboring at least one nonsilent mutation in *CDH10* (n = 12) and those not (n = 84).p* = log-rank test; p = Cox PH regression model; HR = hazard ratio

To evaluate the impact of these mutations in these three genes on patient outcomes, we used a Cox proportion hazard (PH) regression model to correlate the mutation status with survival. The patients were split into two groups: those harboring at least one nonsilent somatic mutation and those without. Among these three genes, patients with mutations in *CDH10*, a cadherin which is predominantly expressed in brain [11], displayed a significant association with poor survival, after adjusting for age, gender, tumor stage, and chemotherapy status (p = 0.0127). Twelve of 99 patient harbored *CDH10* mutations, mostly located in the cadherin domain with high confidence protein affecting predictions (i.e. SIFT) (**Fig 1**).

**Fig 2:**
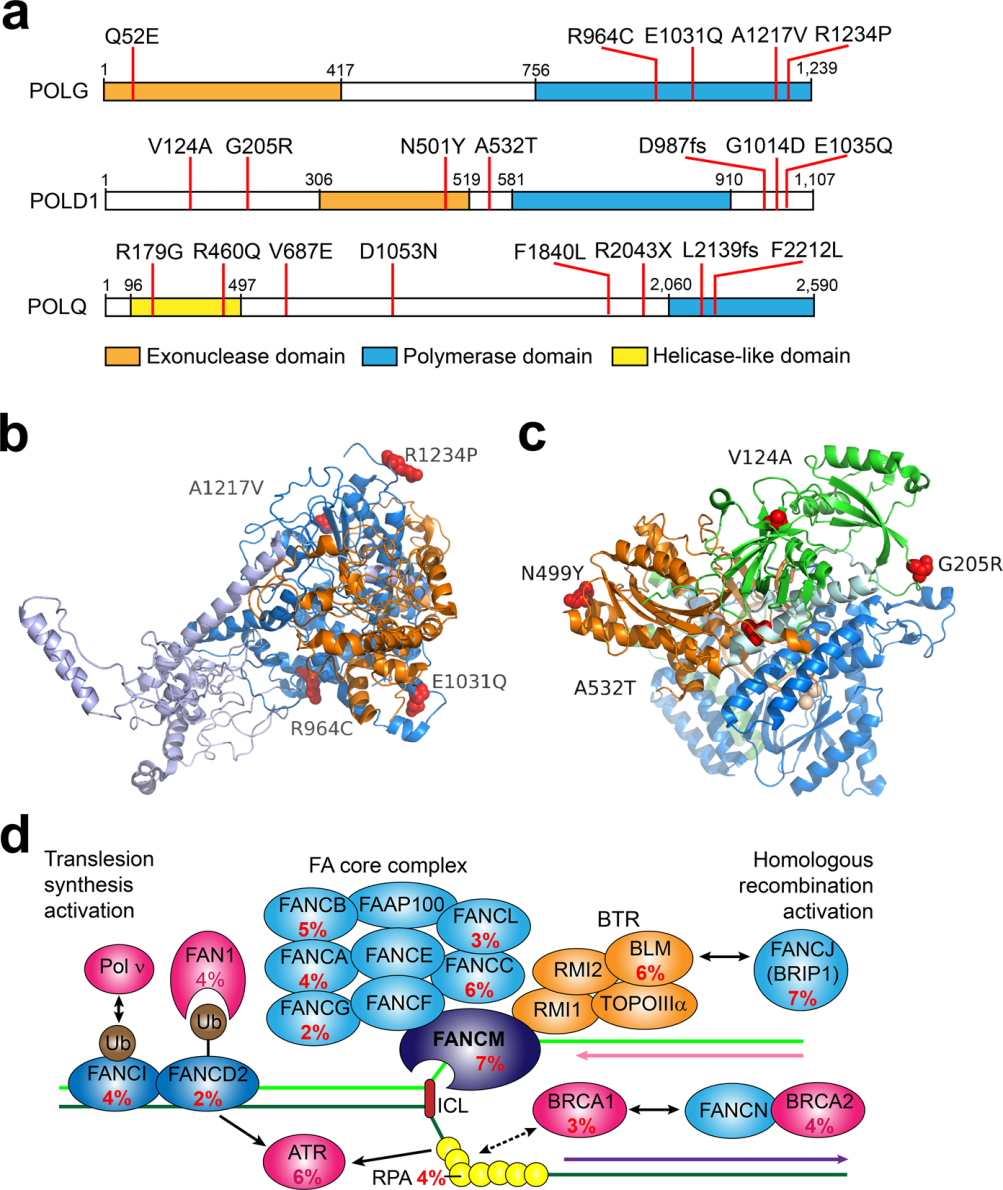
Top mutated DNA polymerases and mutation prevalence in Fanconi anemia pathway genes in SCLC. **a**) Schematic representation of amino acid changes in human POLG, POLD1, POLQ proteins; **b**) the amino acid alterations in human POLG catalytic domain. Mutations were mapped onto the structure of human POLG using PDB Id entry 3IKM as template [6]. **c**) Relevant amino acid alterations in POLD1. Mutations in human POLD1 gene were mapped onto structure of the yeast DNA polymerase subunit δ using PDB entry 3IAY Orange colored ribbon represents exonuclease domain, blue colored ribbon corresponds to polymerase domain, and the green ribbon represents the N-terminal portion of the protein [27]. The mutations in both structures are shown in red spheres. **d**) Mutation prevalence in Fanconi anemia pathway genes.

To better understand the genetic basis of chemo sensitivity and resistance in SCLC, we systematically surveyed SNVs and indels in all known DNA repair genes [12]. Eighty-seven percent (87%) of patients harbored ≥1 nonsilent somatic SNV in a DNA repair gene besides *TP53* (**S4 Table**); similarly, within a Japanese SCLC study cohort in a previous study, 69% of patients were identified by the same criterion [5]. The patient prevalence of nonsilent somatic SNVs in genes classified as mismatch repair (MMR), nucleotide excision repair (NER), homologous recombination, or DNA polymerase were 22%, 30%, 26% and 35%, respectively. Twelve percent of patients harbored nonsilent somatic SNVs in DNA polymerase genes that are involved in DNA replication in NER and MMR (*POLD1* and *POLE*, [13]). *POD1*, *POLG* and *POLQ* were most recurrently mutated among the 15 DNA polymerase genes. These somatic SNVs cause protein truncations and amino acid changes in the polymerase, exonuclease, and helicase domains (**Fig 2A–2C**). Fanconi anemia pathway genes were most recurrent with prevalence of 36%. Within this specific pathway, multiple genes involved in DNA inter-strand crosslink repair such as *FANCM* (7%) and *BRIP1*/*FANCJ* (7%) were among the most mutated (**Fig 2D**). Finally, 29% of patients harbored nonsilent somatic SNVs in genes that affect sensitivity of mammalian cells to topoisomerase inhibitors, in addition to *TP53* [14].

### Recurrent somatic copy number variants SCLC Chinese patients

**Fig 3:**
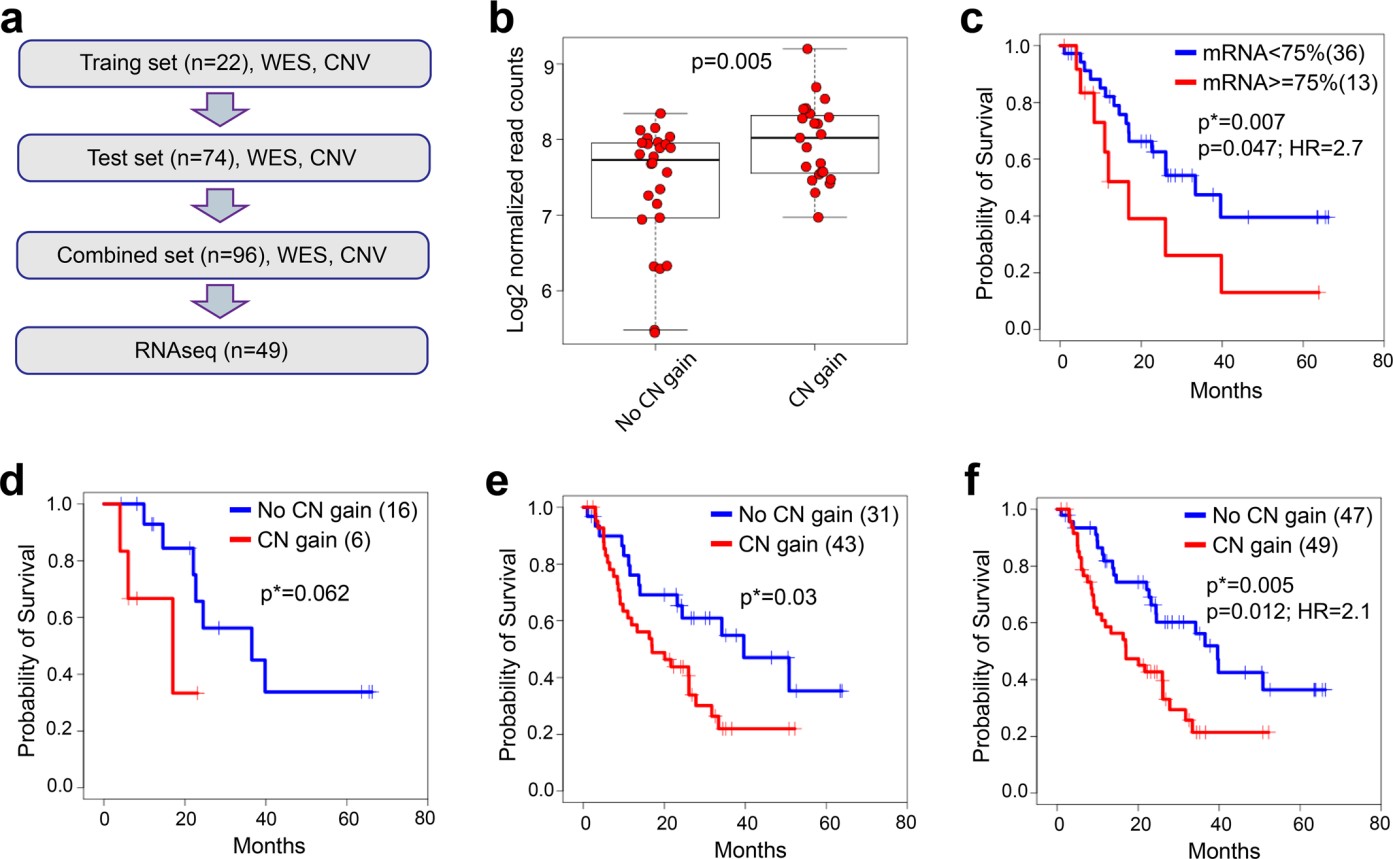
*SRSF1* CN gain and mRNA expression correlates with survival. A: The time-to-event analysis schema with available patient specimens. In the time-to-event analyses, 96 Chinese primary SCLC patients with clinical outcome were divided into training and test cohorts according to the availabilities of matched normal, RNAseq and survival outcome information. The training set includes 22 patients with each patient having tumor and normal WES data and survival outcome. The test set includes 74 patient tumors only. Each patient has WES data from tumor and survival outcome. Among those patients, 48 patients have WES, RNAseq data, and survival outcome. **b**) *SRSF1* mRNA expression in CN gain group and no CN gain group (p = Welch’s t-test). **c**) Kaplan-Meier (KM) curves comparing survival between *SRSF1* low and high mRNA expression groups (n = 48). Similarly, KM curves used to evaluate the difference of survival between different *SRSF1* CN statuses in **d)** discovery set (n = 22), **e**) validation set (n = 74), and **f**) combination of discovery set and validation set (n = 96). p* = log-rank test; p = Cox PH regression model; HR = hazard ratio.

Somatic copy number variants (CNVs) were identified from exome-sequencing data. Our results confirmed key oncogenic genes with recurrent CN gains/amplifications that were previously reported in SCLC [3, 5, 15,16,17], including *MYC* (8%), *KIT* (16%), and *SOX2* (67%). Significant copy number gains or amplifications were observed across a cluster on chromosome 3q26-29 [5] (**S5 Table**). Genes with CN losses previously reported in SCLC [2, 4, 5] include *RB1* (34%), *RASSF1* (57%), *FHIT* (54%), *KIF2A* (16%), and *PTEN* (13%). A long segment along chromosome 3p22 was also detected to have significant CN loss. Recurrence rates of these genes affected by CNVs were comparable to those reported previously [3, 5]. In addition, we found recurrent gains of *SRSF1* (50%) as well as concordant over-expression of mRNA for those patients with gains (p = 0.005; two-tailed two-sided Welch’s t-test; **Fig 3A**). Among these 96 Chinese patients, 28% had both CN gain and mRNA over-expression of *SRSF1*; in an independent cohort of 25 Caucasian SCLC patients (commercially purchased specimens–see Methods), we identified 32% with the same result. Further, *SRSF1* CN gain was determined to be 30% (8/27 SCLC patients) in a re-analysis of the available WES data published from a previous Caucasian SCLC patient cohort–a result very similar between both Caucasian SCLC cohorts [3]. CN gains/amplifications or losses and somatic SNVs for relevant genes are summarized in **S1 Fig**.

*SRSF1* CN status was evaluated by FISH assay (N = 34). Using a FISH criterion described in the **Methods** for deviations from disomy [18], the sensitivity and specificity were 47% and 71% respectively (positive and negative predictive values of 57% and 62%, respectively). This is comparable to a previous study’s concordance reported between FISH and sequencing using much greater sequencing depth (843X) detecting an EML4-ALK fusion in lung cancer [19]. Further, a clinical study detecting ALK fusions in lung cancer reported a positive predictive value between sequencing and FISH as 68% (19/28) among diagnostic characterized patients, and only 46% (6/13) when reduced to those patients with clinical outcomes (11/13 were sequencing positive and partial responders to crizotinib) [20]. These studies support both the lack of sensitivity in FISH assays compared to sequencing for detecting variants and comparability in concordance between these two assays in this study and two previous studies, both of which were detecting a much larger genetic variant (**S6 Table; S2 Fig**).

### *SRSF1* CN gain and mRNA over-expression predicts poor survival in Chinese SCLC patients

For patients with both survival and WES data (N = 96), genes within CN gain or loss regions were correlated with survival. The cohorts were separated into a discovery set (patients with tumors/matched normal; N = 22) and a validation set (patients with tumors only; N = 74). Kaplan-Meier analyses were conducted between patients with or without CN gains in the discovery cohort first (see Methods). Then this gene list was reduced to those with log-rank p<0.05 in the validation cohort. For the remaining genes, patients with both RNASeq and survival data were interrogated (N = 48) and *SRSF1* was the only gene that correlated between both CN gain and mRNA over-expression at a p<0.05 (log-rank p = 0.008; **Fig 3B**) as well as between over-expression and survival using a Cox proportion hazard (PH) regression model adjusting for age, gender, tumor stage, and chemotherapy status (p = 0.047; HR = 2.7; **Fig 3C**). Patients with *SRSF1* mRNA over-expression or CN gain demonstrated significantly worse survival. The discovery (log-rank test p = 0.062), validation (log-rank test p = 0.03), and combined patient cohort (Cox PH p = 0.012; HR = 2.1; log-rank test p = 0.005) analyses are provided in **Fig 3D–3F**and **S7 Table** CN gains in *SRSF1* from The Cancer Genome Atlas (TCGA) were interrogated for correlation with survival (**S3 Fig** to evaluate the specificity of *SRSF1* CN gains associating with survival in other cancer indications. We used a threshold of at least 3 patients for a particular cancer indication harboring a CN gain in *SRSF1* to minimize biases in sample groups for survival analysis. Among cancer indications in TCGA with ≥3 patients harboring CN gains in *SRSF1* (BRCA, KIRP, SARC, SKCM, and UCEC), uterine corpus endometrial carcinoma (UCEC) was the only indication with a correlation between *SRSF1* CN gain and poor survival (log-rank test p = 0.003), though the patient number with a CN gain group was highly unbalanced compared to those without (n = 8 vs. n = 437, respectively), likely driving the low p-value. This result demonstrates how this CN gain in *SRSF1* is specific to SCLC.

### *SRSF1* is a key mediator of growth and survival in *SRSF1* high-expressing SCLC

**Fig 4:**
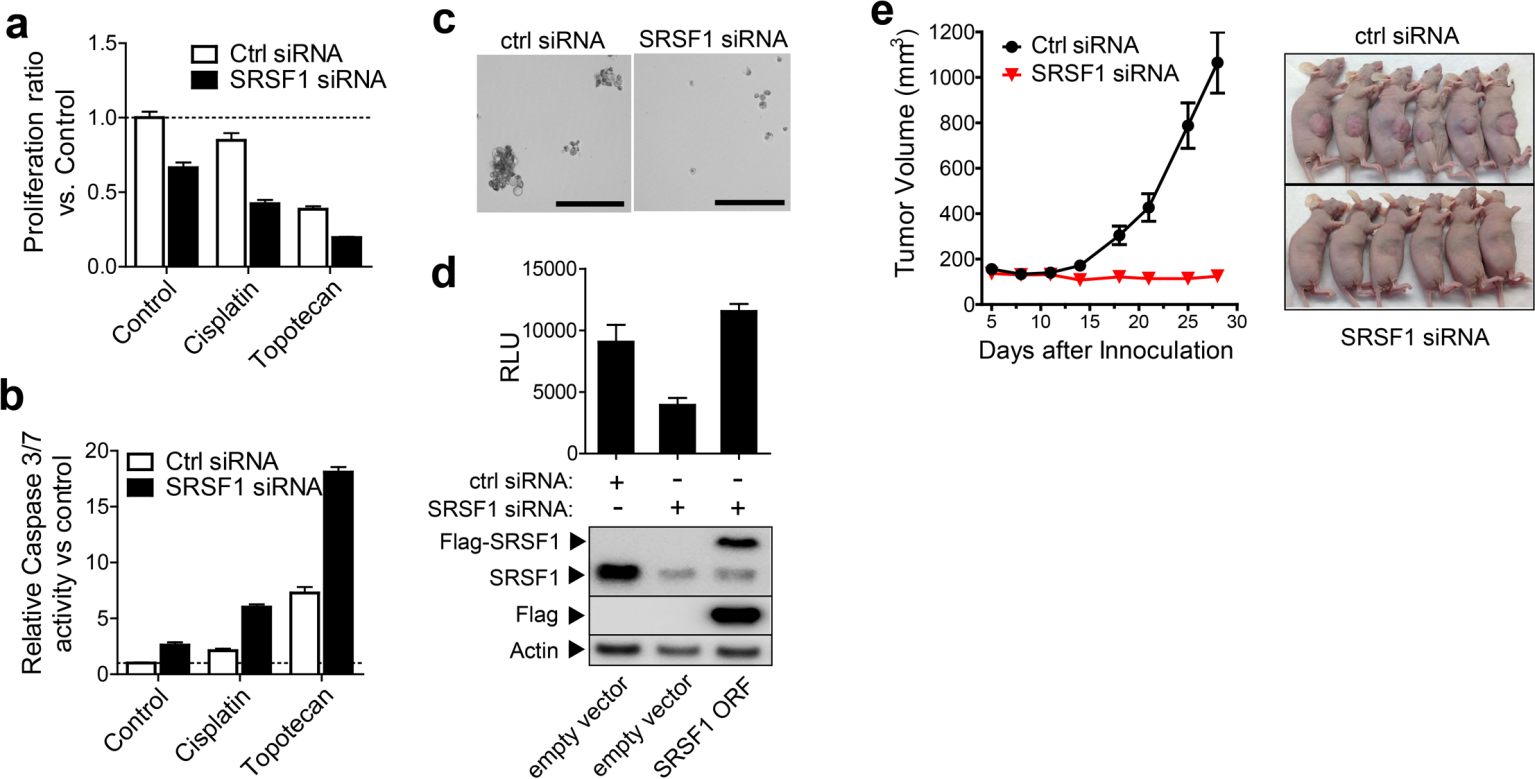
SRSF1 is required for tumorigenecity of SCLC. (**a**) and (**b**): DMS114 cells were transfected with non-targeting control or SRSF1-directed siRNAs for 48 hrs, then treated with cisplatin (2.5ug/ml) or topotecan (2.5ug/ml) for 24 hrs. Cell growth (**a**) and Caspase-3/7 activities (**b**) were assessed and normalized against non-targeting ctrl siRNA-transfected cells as 100% control. (**c**): DMS114 cells were transfected with non-targeting and SRSF1 siRNAs for 48 hrs and then seeded in sphere forming media and allowed to grow for 4 days. Phase-contrast images of the sphere formation under each condition were captured and viable cell mass quantitated by CTG assay. (**d**): Reconstitution of SRSF1 expression using a siRNA-resistant Flag-tagged SRSF1 expression construct was carried out in SRSF1 siRNA transfected cells. Impact on sphere growth rate was assessed by CTG assay, and successful SRSF1 protein re-expression was confirmed using either anti-SRSF1 antibody or anti-Flag antibody. (**e**) DMS114 cells transfected with non-targeting control siRNA or SRSF1 siRNA were implanted into immunocompromised mice and tumor formation rates were monitored and measured as described in Materials and Methods.

We next evaluated SRSF1 as a potential tumor driver in SCLC. We first screened *SRSF1* DNA CNs in 13 SCLC cell lines using TaqMan assays. Five of thirteen had *SRSF1* CN> = 3: Four including NCI-H82 had 3 copies, and DMS114 had 4 copies. These cell lines also expressed high levels of *SRSF1* protein (**S4 Fig**). *SRSF1* siRNA was transfected into DMS114, and the growth effect of *SRSF1* ablation in two dimensional cell culture either alone or in conjunction with a sub-lethal dose of cisplatin or topotecan (two of the most common standard of care treatments in SCLC), was evaluated (**Fig 4A**). SRSF1 knockdown alone caused a 35% decrease in the proliferation rate. Treatment with a low dose of cisplatin or topotecan only induced a modest decrease of cell growth. However, combination with *SRSF1* siRNA significantly enhanced the overall growth inhibition effect.

SRSF1 has also been shown to regulate the BCL2 pathway by alternative splicing of BIM, which results in a protein lacking pro-apoptotic activity [21, 22]. In this study, we see that *SRSF1* gene expression is positively correlated with BIM (r = 0.58, p<0.0001) and *SRSF1* CN gain or amplification also shows concordantly high expression of BIM (**S5 Fig**). Furthermore, we performed caspase-3/7 assays on similarly treated cells (**Fig 4B**) to evaluate the synergistic effect between SRSF1 knockdown and standard chemotherapy. *SRSF1* siRNA alone induced modest but statistically significant caspase-3 activation, similar to cisplatin treatment alone. The combination of the two produced a substantially higher caspase induction. A similar trend was revealed with topotecan. Comparable results were also obtained in other SCLC models (**S4 Fig**).

The effect of *SRSF1* knockdown on SCLC cells when grown as 3D spheroids was evaluated next. Cells transfected with non-targeting siRNA produced large and well-organized spheroids; in contrast, cells transfected with *SRSF1* siRNA did not form well-organized structures but mainly existed as single cells with poor viability (**Fig 4C** and **S6B Fig**). Results were confirmed by colony formation assays (**S6D Fig**). The effect of *SRSF1* siRNA is mediated by specific target loss as demonstrated by a reconstitution study with a siRNA-resistant Flag-tagged expression construct which efficiently rescued the spheroid growth in the presence of the *SRSF1* siRNA (**Fig 4D**). A similar rescue effect was also achieved in NCI-H82 cells (**S6C Fig**).

### *SRSF1* is required for in vivo tumorigenicity of SCLC

A tumor formation study was conducted using siRNA-transfected DMS114 and SHP-77 cells. Equal numbers of viable transfected cells were injected in immunocompromised mice and tumor growth was monitored for up to three weeks. *SRSF1* knockdown completely suppressed the tumor growth in both SCLC models (**Fig 4E** and **S7A Fig**).

### *SRSF1* silencing triggers DNA-damage and suppresses *PI3K/AKT* and *MEK/ERK* pathways

**Fig 5:**
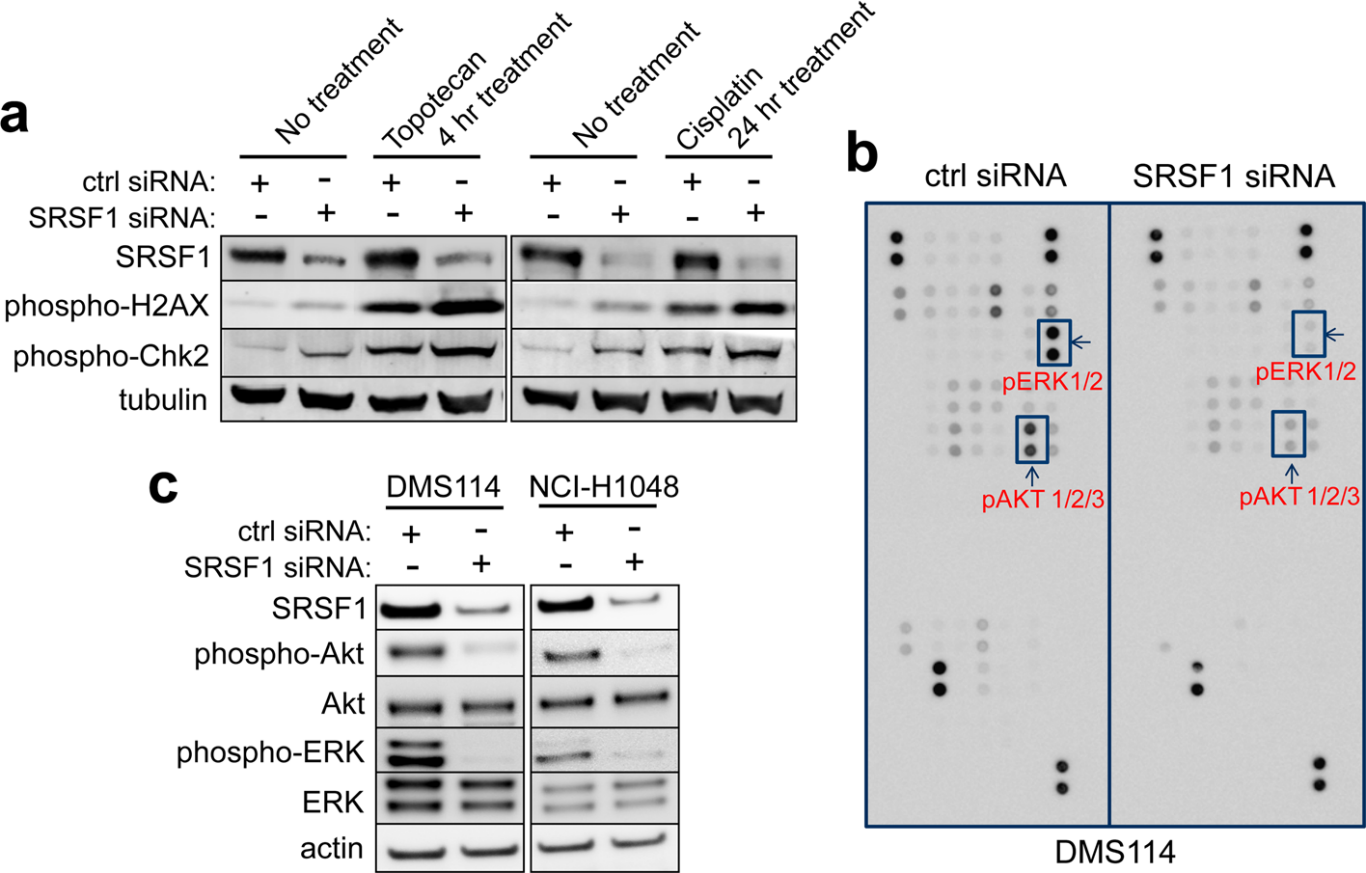
Mechanism of action for SRSF1 in SCLC. **a**) SRSF1 prevents DNA-damage. DMS114 cells were transfected with control or SRSF1 siRNA and then treated with topotecan or Cisplatin for the indicated times. SRSF1, phosphor-H2AX and phosphor-Chk2 were probed with their corresponding antibodies. **b**) **c**) SRSF1 mediates the activation of AKT and ERK pathways. DMS114 cells transfected with ctrl or SRSF1 siRNAs were lysed and applied to the phospho-kinase array as detailed in Materials and Methods. The dot blot result was further confirmed by western blot in both DMS114 and NCI-H1048 cells.

DNA-damage induction as a potential effect of *SRSF1* knockdown based on our DNA-repair analysis was assessed. Inductions of p-H2AX and Chk2, established markers of DNA-strand breaks and DNA-repair response [23, 24], were consistently observed upon SRSF1 abrogation in DMS114 and SHP-77 (**Fig 5A** and **S7B Fig**), and increased phosphorylations were observed when we combined *SRSF1* siRNA transfection and treatment with cisplatin or topotecan.

To better understand the role of *SRSF1* CN gain on downstream pathways in SCLC, we performed differential gene expression analysis between *SRSF1* CN gain and *SRSF1* CN neutral patients. A total of 861 genes were identified to be significantly expressed between these patient cohorts. Pathway analysis revealed that PIK3CA and MAPK3 were two of the top activated master regulators, which suggests that *SRSF1* CN gain regulates PI3K/Akt and MAPK pathway activity with certain causality (**S12 Table**). Therefore, we investigated the impact of SRSF1 loss on both PI3K/Akt and Ras/Raf MAPK kinase signaling pathways in SCLC cells through phospho-kinase array profiling (**Fig 5B**). Control siRNA-transfected DMS114 displayed strong phospho-AKT and ERK signals, which were abrogated by *SRSF1* siRNA. Western blot confirmed this in both DMS114 and NCI-H1048 cells (**Fig 5C**). This demonstrated that SRSF1 promotes SCLC growth and survival by sustaining PI3K/AKT and MEK/ERK pathways, two of the most well-established oncogenic pathways.

## Discussion

Our study represents the first comprehensive genetic landscape survey of Chinese SCLC patients with detailed clinical history, revealing key recurrent genetic alterations associated with patients’ outcomes.

Mutations identified in previous SCLC genomic studies shared little consensus for significantly mutated genes other than *TP53* and *RB1*. However, by leveraging our data with these previous SCLC studies, we were able to identify three additional common significantly mutated genes *(TMEM132D*, *NCAM2*, and *CDH10*) with over 10% prevalence in SCLC. Interestingly, all three genes encode transmembrane proteins involved in neural cell adhesion. This finding will need to be further evaluated for the impact on neuroendocrine association in SCLC.

Cadherins (CDHs) are important in maintenance of cell adhesion and polarity, alterations of which contribute to tumorigenesis. Recurrent mutations in *CDH10* have recently been reported in *EGFR/KRAS/ALK* mutation-negative lung adenocarcinoma in never-smokers [25] and as a prognostic mutation signature in colorectal cancer [26]. Our study indicated that *CDH10* is not only the most commonly and significantly mutated gene in SCLC but also associated with poor survival in SCLC. CDH1/E-cadherin, the founding member of the CDH/cadherin family, undergoes loss-of-function mutations across multiple tumor types such as breast, gastric, colorectal and ovarian cancer. Its functional inactivation contributes to cancer progression by increasing cell invasion, migration, metastasis and proliferation and EMT process [27]. We speculate that the recurrent CDH10 mutations we detected in SCLC may perform similar roles as CDH1 mutations in other cancers to promote SCLC aggressiveness, leading to poor patient survival. We are currently conducting experiments to test this hypothesis.

Our study suggests that genetic alteration of DNA repair pathways influence chemotherapy outcomes in SCLC patients. The Fanconi anemia (FA) pathway is essential for the repair of DNA inter-strand cross-linking agents, such as cisplatin, which has been used as first-line treatment in SCLC. It was demonstrated several decades ago that the FA patient-derived cells which contain genetic defects in FA genes display hypersensitivity to DNA cross-linking agents [28]. Our data strongly suggest that high prevalence mutations in FA pathway genes may contribute to initial hypersensitivity of SCLC to platinum-based treatment such as cisplatin. Multiple reports with experimental evidence show that the efficacy of various chemotherapeutic agents, including cisplatin, requires a functional TP53 protein for efficient induction of apoptosis and that loss of TP53 function enhances resistance to cytotoxic agents used in cancer therapy [29,30,31]. Further, a combination of TP53 inactivation and MMR deficiency has also been observed to confer cisplatin resistance [32]. Our data suggest that high frequency mutations in *TP53* combined with other DNA repair mutations such as mismatch repair, nucleotide excision repair, homologous recombination, and key DNA polymerases may confer early sensitivity and latent resistance to cisplatin in SCLC.

Of particular importance is our discovery of the prevalence of *SRSF1* CN gain and mRNA over-expression, and its role as a prognostic marker for poor patient survival—reported for the first time in SCLC. *SRSF1* occurs in the same protein complex with topoisomerase 1 (*Top1*) [33]. Topotecan is a *Top1* inhibitor and the only agent with regulatory approval for the treatment of relapsed SCLC [34]. In normal cells, Top1 cooperates with SRSF1 to prevent the formation of DNA-RNA hybrids (R-loops), unscheduled replication fork arrest, and genomic instability. In *Top1* deficient cells, R-loops are formed and lead to replication fork stalling, phosphorylation of H2AX, and genomic instability. Treatment of *Top1+* cells with diospyrin, to inhibit *Top1*phosphorylation of SRSF1 or with a siRNA targeting SRSF1 mimics a Top1-deficient phenotype [35]. Although significant correlation between *SRSF1* and *Top1* gene expression is not observed in our data, our experiment clearly demonstrates that SRSF1 loss induces phosphorylated H2AX signal in SCLC cell lines, which suggests that SRSF1 may help maintain the genomic integrity of SCLC to safeguard against DNA-damage and cell death. With these factors in mind, we propose that SRSF1 may also rely on modulating H2AX signal to sustain the tumorigenicity in some SCLC tumor patients.

In the absence of specific limited stage (LS) or extensive stage (ES) disease determination in this study and a recent comprehensive SCLC study [9], a simplified approach was used to classify SCLC patients into early and late stage disease activity. Based on known TNM information, early stage (TNM stage I/II) patients are M0, who are usually designated as LS patients, while late stage (TNM stage III/IV) patients are M1a or M1b, and usually classified into ES patients. We then evaluated *SRSF1* expression between early (TNM stage I/II) and late stage (TNM stage III/IV) SCLC patients. Results indicated that *SRSF1* gene expression does not significantly differ between these patient groups in both this study and the George et al study (p = 0.81 and p = 0.91, respectively; **S8 Fig**). This may suggest that SRSF1 is not the key driver of cancer metastasis in SCLC.

SRSF1 is one of the critical downstream transcriptional targets of Myc [36]. Myc family genes (*MYC* and *MYCN*) were shown to have significant CN gain or amplification events in our Chinese SCLC patients (14%). SRSF1 gene over-expression in both Myc and N-Myc amplified SCLC cell lines and Myc amplified SCLC tumor patients, however, was not observed (p = 0.29 and p = 0.33, respectively), though the number of amplified cell lines or patient tumors with available gene expression data was sparse for each comparison (**S9 Fig**).

*SRSF1* is a key cancer driver, as demonstrated by the profound tumor-suppressive effect of specific *SRSF1* knockdown in *SRSF1*-amplified or overexpressed SCLC models. Previous reports demonstrate that overexpression of *SRSF1* results in oncogenic transformation of immortalized rodent fibroblasts [37], human mammary epithelial cells [38] and mouse hepatocytes [39]. In these models, SRSF1 overexpression promoted cell proliferation, resistance to apoptosis, and formed tumors in orthotopic mouse models. It is likely that this transformation is a cumulative result of SRSF1’s many different functions, including a combination of several alternatively spliced oncogenic variants in response to an increase in SRSF1 levels. A number of such variants have been identified, but these probably represent only a small fraction of potential effectors [40]. Das et al, previously summarized various spliced products of SRSF1 and isoform mechanisms driving oncogenic phenotypes [40], though these were not detected with reliability using RNASeq here–a challenge with this technology that currently persists in splice variant detection, especially in FFEE specimens. Furthermore, we demonstrate here that SRSF1 mediates the activation of both PI3K/AKT and MEK/ERK pathways as evidenced by both gene expression pathway analyses and the suppression of these pathways through SRSF1 knockdown. It is interesting to note that several SRSF1-regulated targets involved in regulating cell proliferation are downstream of these two pathways, including RPS6KB1, MKNK2, and CCND1 genes [37, 41]. *RPS6KB1* encodes the protein S6 kinase 1, a downstream effector in the PI3K/AKT/mTOR signaling pathway and has been shown to be involved in mediating SRSF1-induced transformation [37, 42]. *MKNK2* is an effector in the MAPK/ERK pathway [43]. Splicing functionality has been shown to be critical for some, but not all oncogenic activities of SRSF1. An SRSF1 variant that is confined to the nucleus has been shown to be critical for its oncogenic role in mammary epithelial cells [38]. However, this variant was not able to promote tumor formation in hepatocellular xenografts [39]. In this particular model, SRSF1-mediated oncogenesis was attributed to activation of Raf-MEK-ERK pathway [39]. This demonstrates that SRSF1 can be oncogenic via both nuclear and cytosolic activities through either canonical (splicing-related) or non-canonical (AKT/ERK-related) pathways under various cellular contexts. It may be of future interest to explore and pinpoint which effector pathway of SRSF1 drives its oncogenic roles in SCLCs. In conclusion, our discovery firmly establishes SRSF1 as a compelling therapeutic target for SCLC, especially for the population with poor outcome, as predicted by *SRSF1* over expression.

## Methods

### SCLC patient and sample summary

The study protocol and informed consent from all studies in this study were approved by the Ethics Committee of Shanghai Chest Hospital and Nanjing Medical University. Informed consent in writing was obtained from each patient and the study protocol conformed to the ethical guidelines of the 1975 Declaration of Helsinki as reflected in a priori approval by the Ethics Committee of Shanghai Chest Hospital and Nanjing Medical University.

Ninety-nine Chinese patients who were diagnosed with primary SCLC were recruited prospectively into an ongoing study at the Jiangsu Cancer Hospital or Shanghai Chest Hospital from July 2004 to July 2013. The diagnosis of SCLC was made by pathologists in the above hospitals by hematoxylin and eosin (H&E) staining according to histology plus the immunohistochemistry for chromogranin A and synaptophysin. Patients were followed up prospectively via routine hospital visits or telephone calls. The phone calls were conducted by trained medical staff to patients or their family contacts once every three month until death or last time of follow-up. All patients were treated with at least one cycle of chemotherapy after surgery. The clinical features of the patients are summarized in **Table 1**and **S1 Table**. Of the 99 patients, 25 had matched normal adjacent tissue or blood, while 74 patients only had tumor specimens. All tissues samples were FFPE archived samples collected from surgery (not biopsy). Eighty-six tumor samples were treatment naïve and 13 of 99 patients were treated with standard chemotherapy before surgery. Tumor contents in each tumor and normal adjacent tissue (NAT) was assessed by H&E stain and the tumor and NAT were subjected to macro-dissection and tumor purity was >70%; the tumor content in each NAT was< 3%.

The Caucasian SCLC patient cohort consisted of 25 FFPE lung tumor tissue specimens with matched normal adjacent tissue pairs, which were purchased from Conversant Biologics, Inc (Huntsville, AL) (**S11 Table)**. The diagnosis of SCLC was confirmed by two independent pathologists in Medimmune by H&E staining. All samples were treatment naïve surgical samples. All patients were Caucasian with 24 males and 1 female. The average age of the patients was 63.3 years (range of 40–76 years). The tumor stages ranged from stage I to IV. The tumor and NAT were macro-dissected and tumor purity was >70%; the tumor content in each NAT was< 3%.

### DNA sequence read mapping and variant calling

DNA whole exome sequence (WES) and RNA sequencing data (RNASeq) data was generated using the Illumina standard library preparation and sequencing protocols as described in [44] The SureSelect Human All Exon V5 capture kit was used to capture coding regions of genes included in the major genomic databases. Paired end FASTQ files of 90mer sequence reads for both sequence data types were provided to MedImmune. RNASeq data has been deposited into GEO under accession GSE60052 while WES data was deposited into dBGaP under accession 12059.

All sequence data was QCd for read counts, quality values, kmer usage, GC-content, and all other relevant parameters with FastQC (v0.10.1). The DNA read sequences were aligned to the human genome (UCSC hg19; Feb 2009 release; Genome Reference Consortium GRCh37) using GATK (v2.3.4; [45]) and both insertion/deletion (indel) realignment and PCR duplicate removal was conducted using GATK (v2.3.4; [45]) and Picard (v1.85; [46]) respectively. Both coverage and depth statistics for all 99 tumor specimens are provided in **S10 Table.**

For the 25 tumor/normal matched Chinese and 25 tumor/normal matched Caucasian (commercially purchased) specimens, both Mutect (v1.1.4; [47]) and SAMtools (v0.1.18; [48]) were used to make somatic variant calls. SAMtools mpileup arguments: Qphred>30 and mapping quality>30 with minimum coverage >20; MuTect arguments: default settings. GATK SomaticIndelDetector with default settings and SAMtools mpileup were used to identify small indels. The SNVs and indels which were in common between GATK and Samtools were retained. SNVs and indels were further filtered by 1000 genomes and NHLBI-ESP project with 6500 exomes minor allele frequency (MAF) in all races of <1% or unknown MAF. The retained SNVs/indels were further filtered by dbSNP129 and dbSNP135, following known issues between the two dbSNP versions. Finally, genes were removed from the SNV/indel list that had been identified from a previous study as potential artifact genes, to further minimize false positive variant calls [49] All dbSNPs which were retained in dbSNP135 and had Cosmic IDs were noted for further study.

For the 74 DNA tumor specimens without a matched normal specimen, Samtools mpileup was used to call SNVs and indels relative to the human reference genome (UCSC hg19; Feb 2009 release; Genome Reference Consortium GRCh37). Germline polymorphisms were removed by retaining only mutations with MAF in all races of <1% or unknown MAF within the 1000 genomes and NHLBI-ESP project with 6500 exomes database. The retained SNVs/indels were further filtered by dbSNP129 and dbSNP135 similar to previously described. The most recurrent SNVs/indels between the matched and unmatched patient cohorts are provided in **S2 Table**, along with patient recurrence summaries from a previous Japanese SCLC cohort of 51 patients, to highlight comparability in results and a validation of the SNV/indel calling strategy [5]. A similar strategy for calling and filtering somatic SNVs in the absence of a matched germline control specimen was conducted in a previous prostate cancer whole exome study [50]. All patient-level somatic SNV or indel calls with associated read depth and annotation parameters are provided in **S8 Table**.

SNV and indel annotation was conducted with ANNOVAR [51]

### Patient identity QC

To verify the identity and matching between the tumor and normal paired WES samples, a selection of 300 heterozygous single nucleotide polymorphisms (SNPs) with MAFs>0.3 and <0.7 were selected from the 1000 genomes database. All DNA samples were clustered to observe any major discrepancies in subject or specimen labeling (**S10 Fig**).

### Recurrent driver gene identification

All somatic mutations in the coding regions (plus splicing mutations) were selected for driver gene prediction analysis to identify those genes with the most recurrent nonsilent mutations. MutsigCV [39] and the method described by Youn et al [52] were implemented independently and Q value<0.05(MutsigCV) and Q value = 0.00 (Youn’s method) were used as thresholds to detect significantly recurrently mutated genes. Genes predicted by both methods were selected as high confidence driver genes (**S3 Table)**.

### DNA polymerases structure modeling

Amino acid change mutations were mapped onto corresponding structures using mutagenesis wizard implemented in PyMOL (Schrodinger, LLC). For POLG coordinates of human mitochondrial DNA polymerase holoenzyme from Protein Data Bank (PDB, [53]) entry 3IKM [54] were used. The Q52E mutation could not be mapped since that part of the protein was absent in the structure. For DNA polymerase delta subunit the PDB entry 3IAY of yeast that shares 48/65% sequence identity/similarity over 908 amino acids was used.

### RNA sequence read mapping and differential expression analysis

For RNASeq data, the average read count per mate was 50 million. RNA reads were mapped to the human genome (UCSC hg19; Feb 2009 release; Genome Reference Consortium GRCh37) using TopHat2 (v2.0.9; [55, 56]) and the human reference gtf annotation file (GRCh37.68). Transcript counts were calculated and normalized using htseq-count and DESeq (v1.12.1; [57]). The DESeq negative binomial distribution was used to calculate the p-value and fold changes between 48 lung tumor and 6 normal adjacent lung samples using adjusted p<0.05 and |fold change|>2 as a threshold. The full transcriptome summary table is provided (**S9 Table**). Due to the low fidelity and lack of reproducibility in splice variant detection using RNASeq, analysis was not conducted to examine spliced products of SRSF1.

### Somatic copy number variation (CNV) analysis

For CNV analysis, the R package ExomeCNV [58] was used. This method makes CNV calls not by defining a mandatory cut-off to detect gains or losses, rather the specificity and sensitivity (power) of detecting CNV based on depth of coverage and log ratio of all exons is calculated, and a CN call is made when sufficient specificity and sensitivity are achieved. We used default parameters setting of ExomeCNV (sensitivity and specificity = 99.9%). For the 22 tumor/normal matched Chinese as well as the 25 tumor/normal Caucasian (commercially purchased) specimens, the standard ExomeCNV pipeline was employed, in which a tumor and its adjacent normal pair were used to make the call. For the 74 tumor specimens without matched normal tissue, 1 normal FFPE lung tissue specimen (N08-4579A) was used as baseline with each of the 74 tumor specimens using ExomeCNV. This method was also conducted with 6 normal FFPE lung tissue specimens and results were very similar between the use of a single normal or average of 6 normals. The overview of the most prevalent CNV calls (≥20% patients harboring gains or losses, to limit the table size) for matched Chinese patient tumor/normal or Chinese patient tumor only results are provided in **S5 Table**.

### TCGA data to evaluate SRSF1 CN gain correlation with survival in other indications

All cancer indications in TCGA were assessed for correlation with survival using OncoLand (OmicSoft Corp; Cary, NC). To avoid issues of unbalanced comparisons, only indications where at least 3 patients harboring a CN gain in SRSF1 were analyzed. These included: breast invasive carcinoma (BRCA), kidney renal papillary cell carcinoma (KIRP), sarcoma (SARC), skin cutaneous melanoma (SKCM), and uterine corpus endometrioid carcinoma (UCEC). UCEC was the only indication with a correlation between patients harboring CN gain of SRSF1 and poor survival (log-rank test p = 0.003), though the number of patients harboring a CN gain was highly unbalanced compared to those without (n = 8 vs. n = 437, respectively; **S3 Fig**).

### Time-to-event analyses

Time-to-event analyses were used to correlate both the CN gain status of *SRSF1* and *SRSF1* gene expression with overall survival of Chinese SCLC patients. First, a Kaplan-Meier (KM) analysis was used to evaluate the difference of survival curves for *SRSF1* CN gain group and no CN gain group. Those genes with a trend of significance (log-rank p<0.1) in the Chinese patient discovery cohort (n = 22; *SRSF1* in **Fig 3D**) and with 10% CNV calls among the cohort were evaluated in the Chinese patient validation cohort (n = 74; 1,707 genes; *SRSF1* in **Fig 3E**). Since the discovery cohort was approximately 1/3 the size of the validation cohort and thus less powered, a modest log-rank test threshold was used. Among those 1,707 genes, 215 had p-values<0.05 from the log-rank test and CNV calls in more than 10% of the patients in the cohort. Among these 215 genes, *SRSF1* was the only gene that correlated with DNA CN gain status using a Welch’s modified t-test (p<0.01; **Fig 3B**).

Next, both the Chinese patient discovery and validation cohorts were combined (n = 96) and both a KM and multivariate Cox proportion hazard (PH) regression analysis was conducted to compare the *SRSF1* CN gain and no CN gain patient groups. Differences were assessed with p-values for the grouping difference (log-rank) and the hazard ratio with adjustment for age, gender, tumor stage and chemotherapy treatment status before sampling (Cox PH model; **Fig 3F**).

Then, the gene expression of *SRSF1* in the 48 Chinese SCLC patients with RNASeq and clinical data were divided into two groups according to *SRSF1* gene expression level (>75% percentile of overall expression and < = 75% percentile of overall expression). Similar KM analysis as well as a Cox PH regression analysis was performed to compare the survival curves of *SRSF1* over- expressed versus not over-expressed groups with the same covariate adjustments in the Cox PH model as conducted previously with WES data (**Fig 3C**). The R package survival was used to perform these analyses and model summaries are provided in both **Fig 3** and **S7A and S7B Table**.

A similar time-to-event analysis adjusting for age, gender, tumor stage and chemotherapy treatment status was conducted using the nonsilent mutation status to split patients into two groups.

### FISH confirmation of SRSF1 CN gain status

SRSF1 gene copy number change was conducted via a dual-probe FISH test. The SRSF1 FISH probe was a SpectrumRed (Cat #02N34-050, Enzo Life Sciences, Inc., New York, USA) labeled fluorescent DNA probe, generated in-house from a bacterial artificial clone CTD-2061E5 (Invitrogen, Carlsbad, USA). CEP17 probe (Vysis, Cat #06J37-017) was a SpectrumGreen labeled fluorescent DNA probe specific for the alpha satellite DNA sequence at the centromeric region of chromosome 17.

FISH assays were performed as reported previously. In brief, assays were run on 4 micron dewaxed and dehydrated FFPE samples from 34 small cell lung cancer patients. The SpotLight Tissue pretreatment Kit (Cat #00–8401, Invitrogen, Carlsbad, USA) was used for pretreatment according to the manufacturer’s instructions. Sections and probes were codenaturated at 79oC for 6 minutes and then hybridized at 37oC for 48 hours. After a quick post wash off process (0.3%NP40/2xSSC at 75.5 oC for 2 minutes, twice in 2×SSC at room temperature for 2 minutes), sections were finally mounted with 0.3μg/ml DAPI (Cat #H-1200, Vector Laboratories, Inc., Burlingame, USA).

CN gains were scored using the criteria outlined by Cappuzzo et al (18) where disomy was scored by ≤2 copies in ≥90% of cells, low trisomy was scored by ≤2 copies in ≥40% of cells and ≥3 copies in 10–40% of the cells, high trisomy was scored by ≤2 copies in ≥40% of the cells and ≥3 copies in ≥40% of the cells, and polysomy was scored by ≤2 copies in <40% of the cells. High trisomy and polysomy were called CN gain positive. (**S6 Table**).

### Taqman assay for SRSF1 CNV status in SCLC cell lines

Genomic DNA (gDNA) from cultured cells was prepared using QIAamp DNA Micro Kit. Copy number assay of SRSF1 (Hs00944074_cn) and reference assay RNase P (VIC) were ordered from ABI/Life Technologies. Assays were set up based on ABI reference with four replicates for each sample. The assays were run on ABI 7900HT (SDS v2.X) and the data files were analyzed using the CopyCaller Software. Reference probe RNAse-P was used to determine the SRSF1 copy number gain status: copy number > 2 was considered a gain status.

### Cell culture, antibodies, and function assays

All SCLC cell lines were grown in RPMI1640 medium supplemented with 10% fetal bovine serum. SRSF1 (SF2/ASF) antibody (96) was supplied by Santa Cruz Biotechnology. Phospho-Histone H2A.X (Ser139) (20E3) and Phospho-Chk2 (Thr68) (C13C1) were supplied by Cell Signaling Technology. Cell proliferation was determined by CellTiter-Glo Luminescent Cell Viability Assay (Promega). Caspase-Glo 3/7 Assay Systems (Promega) were used to analyze cell apoptosis.

### siRNA transfection

SiRNA reverse transfections were carried out using Lipofectamine RNAiMAX (Life Technologies). siRNAs targeting SRSF1 were ordered as “HP custom siRNA” from Qiagen. The sequences is and CCAACAAGATAGAGTATAA (SRSF1 siRNA). AllStars Neg. Control siRNA (Qiagen) was used as negative control for transfection. Both control siRNA and SRSF1 siRNAs were transfected at a final concentration of 100nM. Culture medium were was replaced with fresh medium at 48 hour after transfection, and cell lysates were prepared at 72 hour for Western blotting.

### Colony formation assays

For clonogenic assay, SCLC cell lines were transfected with SRSF1 siRNAs for 48 hrs and then seeded in a 1% methylcellulose H4100 medium (StemCell Technologies) consisting of RPMI1640 medium with 10% FBS at 2,000 cells/mL. After 5 days, colonies with more than 40 cells per colony were counted.

### Sphere forming assays

SCLC cell lines were transfected with SRSF1 siRNAs for 48 hrs and then seeded in ultralow attachment plates (Corning) in sphere forming media: DMEM/F12 with 0.4% BSA, 10ng/mL bFGF, 20ng/mL EGF, 5ug/mL insulin, 1% KnockOut Serum Replacement (Life Technologies). Cells were treated with Cisplatin (0.001 ug– 10 ug/ml) for 4 days, after which viability of spheres was quantitated by CellTiter-Glo Assay (Promega). Images were taken with EVOS FL Auto Cell Imaging System.

### SRSF1 rescue assays

SCLC cell lines were cotransfected with 800 ng myc/flag-tagged SRSF1 vector (Origene) encoding the open reading frame of either the wildtype gene (NM_006924.4 with 25 nM of either non-targeting siRNA or SRSF1 siRNA-2 using Lipofectamine RNAiMAX (Life Technologies). SRSF1 siRNA targets the 3’UTR of SRSF1, and therefore does not affect expression of the SRSF1 ORF vector. After 48 hr, cells were harvested and then seeded in ultralow attachment plates (Corning) in sphere forming media: DMEM/F12 with 0.4% BSA, 10ng/mL bFGF, 20ng/mL EGF, 5ug/mL insulin, 1% KnockOut Serum Replacement (Life Technologies). Cells were also harvested and lysed with Novex Tris-Glycine SDS Sample Buffer (Life Technologies) for Western blotting. Viability of spheres was quantitated after 4 days by CellTiter-Glo Assay (Promega). Images were taken with EVOS FL Auto Cell Imaging System

### Xenograft studies in mice

All animal procedures were conducted in accordance with all appropriate regulatory standards under protocols approved by the Medimmune Institutional Animal Care and Use Committee. Since the SRSF1 siRNA had shown good knockdown efficacy of SRSF1 protein at day7 after transient transfection (by western blot of sphere assays), and prolonged effects on colony formation (about 2 weeks after transfection), we used transient siRNA knockdown in the mice xenograft study. Immunocompromised athymic nude (nu/nu) female mice were purchased from Harlon Laboratories at 3–4 week of age. SHP-77 and DMS-114 cells were transfected with either control siRNA or SRSF1 siRNA at a final concentration of 100nM. Two days after transfection, ten million viable cells in 50% matrigel were inoculated subcutaneously (SC) into right flank of each mouse. The length and width of each tumor was measured with an electronic cliper 2 times per week. Tumor growth curves of DMS114 and SHP77 parental cell lines are displayed in **S11 Fig**. Tumor volume (mm3) was calculated based on the following formula: [length (mm) x width (mm)2] ÷ 2.

## Supporting Information

S1 Fig Genomic alterations in Chinese SCLC patients. Tumor samples (n = 99) are ordered from left to right based on SRSF1 copy number gains. Mutations and DNA copy number alterations of key SCLC oncogenic genes are indicated for each sample according to the color legend below the figure. The genomic alteration frequencies for each candidate gene are displayed on the left.
(DOCX)

S2 Fig SRSF1 gene copy number detection by FISH. Representative images show a) SRSF1 normal and b) SRSF1 copy number gain. Red signals represent SRSF1 gene and green signals represent of CEP17; c) SRSF1 CNV SCLC patient prevalence as well as other CNV segments across chromosome 17. Red lines indicate CN gains and green lines indicate CN losses.
(DOCX)

S3 Fig KM curves from indications in TCGA where at least 3 patients harbored a CN gain of SRSF1. Plots were generated in OncoLand (OmicSoft Corp; Cary, NC).
(DOCX)

S4 Fig (a): TaqMan assays of SRSF1 DNA CNs in 13 SCLC cell lines. (b): Western blots of SRSF1 show protein expression levels in SCLC cell lines. (c):NCI-H82, SHP-77 and NCI-H1048 were transfected with non-targeting control or SRSF1-directed siRNAs for 48 hrs, then treated with cisplatin (2.5ug/ml) or topotecan (2.5ug/ml) for 24 hrs. Cell growth and Caspase-3/7 activities were assessed and normalized against ctrl siRNA-transfected cells as 100% control.
(DOCX)

S5 Fig The association of SRSF1 gene expression with BIM gene expression in Chinese SCLC patients (N = 49). The correlation between SRSF1 and BIM gene expression is significant, which likely confirms SRSF1 over expression promotes alternative splicing of BIM.
(DOCX)

S6 Fig NCI-82, SHP-77 and NIH-H1048 cells were transfected with non-targeting and SRSF1 siRNAs respectively for 48 hrs and then seeded in sphere forming media and allowed to grow for 4 days. (a): Phase-contrast images of the sphere formation under each condition were captured. (b): viable cell mass quantitated by CTG assay. (c): Reconstitution of SRSF1 expression using a siRNA-resistant Flag-tagged SRSF1 expression construct was carried out in SRSF1 siRNA transfected NCI-H82 cells. Impact on sphere growth rate was assessed by CTG assay, and successful SRSF1 protein re-expression was confirmed by WB using either anti-SRSF1 antibody or anti-Flag antibody. (d): Clonogenic assays of DMS-114, NCI-82, SHP-77 and NIH-H1049. Cells were transfected with siRNAs for 48 hrs and then seeded in the methylcellulose medium for 7~14 days, colonies with more than 40 cells per colony were counted.
(DOCX)

S7 Fig (a)SHP-77 cells transfected with non-targeting control siRNA or SRSF1 siRNA were implanted into immunocompromised mice and tumor formation rates were monitored and measured. (b): SHP-77 cells were transfected with control or SRSF1 siRNA and then treated with topotecan or Cisplatin for the indicated times. SRSF1, phosphor-H2AX and phosphor-Chk2 were probed with their corresponding antibodies.
(DOCX)

S8 Fig The association of SRSF1 gene expression with early stage and late stage (ES) SCLC patients in both our Chinese SCLC study (left figure) and George et al, study (right figure). P-value is calculated using Welch’s modified t-test.
(DOCX)

S9 Fig The association of SRSF1 gene expression with MYC CNV status. SCLC cell lines (left figure, N = 11) and our Chinese SCLC study (right figure, N = 49 patients with matched expression and CNV samples). P-value is estimated using standard t-test.
(DOCX)

S10 Fig Identity check between matched SCLC tumor and normal specimens. Pearson correlation heatmap is used to compare 300 germline SNP profiles between each of the 25 tumors and matched normals. Gradient of colors: green = no correlation; yellow = low correlation; white = high correlation.
(DOCX)

S11 Fig Tumor growth curves for DMS114 and SHP77 parental cell lines. A total of 5–10 millioins cells were injected to establish xenograft tumors.
(DOCX)

S1 Table Chinese patient clinical summary (n = 99). (XLSX)

S2 Table Recurrent somatic mutated genes from 99 Chinese SCLC patients; additional recurrent rates from 51 SCLC Japanese patients in an independent study [5]. (XLSX)

S3 Table Cancer driver gene intersection by two methods and two public data sets in 99 Chinese SCLC. (XLSX)

S4 Table SNVs within DNA repair mechanisms in Chinese SCLC patients (n = 99) using nonsilent somatic SNVs and indels. (XLSX)

S5 Table Patient counts harboring recurrent (prevalence>20%) gene-level somatic CNVs in discovery and validation cohorts (n = 96). (XLSX)

S6 Table FISH assay confirmation of WES CN calls for SRSF1. (XLSX)

S7 Table A. Kaplan-Meier analysis summary for SRSF1 DNA amplification and mRNA expression; B. Cox proportion hazard regression analysis summary for SRSF1 DNA amplification and mRNA over-expression.
(XLSX)

S8 Table Patient-specific somatic nonsilent SNVs/indels from 99 Chinese SCLC patients. (XLSX)

S9 Table Gene expression (RNASeq) of top up/down regulated genes between 48 tumor and 6 normal samples. (XLSX)

S10 Table WES coverage and depth statistics for 99 tumor specimens. (XLSX)

S11 Table Commercial Caucasian patient information. (XLSX)

S12 Table Mater Regulator Analysis (Ingenuity Pathway Analysis) for differential expression genes between SRSF1 CN Gain / Amp and SRSF1 CN Neutral. (XLSX)
