# Supplementary figures and images for: Genomic Landscape Survey Identifies SRSF1 as a Key Oncodriver in Small Cell Lung Cancer
Source: PLoS Genet. 2016 Apr 19;12(4):e1005895. doi: 10.1371/journal.pgen.1005895 (PMC4836692; doi:10.1371/journal.pgen.1005895)

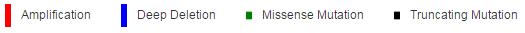


Gain/Amp


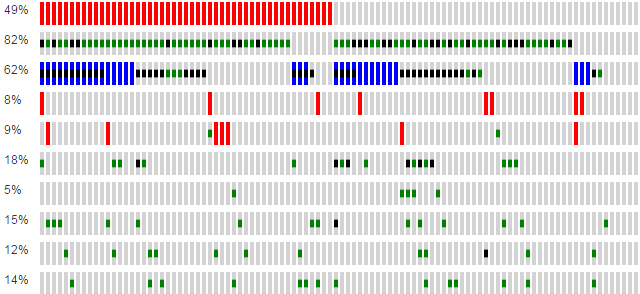

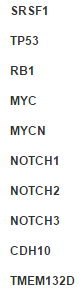

Supplement: S1 Fig — Tumor samples (n = 99) are ordered from left to right based on SRSF1 copy number gains. Mutations and DNA copy number alterations of key SCLC oncogenic genes are indicated for each sample according to the color legend below the figure. The genomic alteration frequencies for each candidate gene are displayed on the left. (DOCX) [file pgen.1005895.s001.docx]

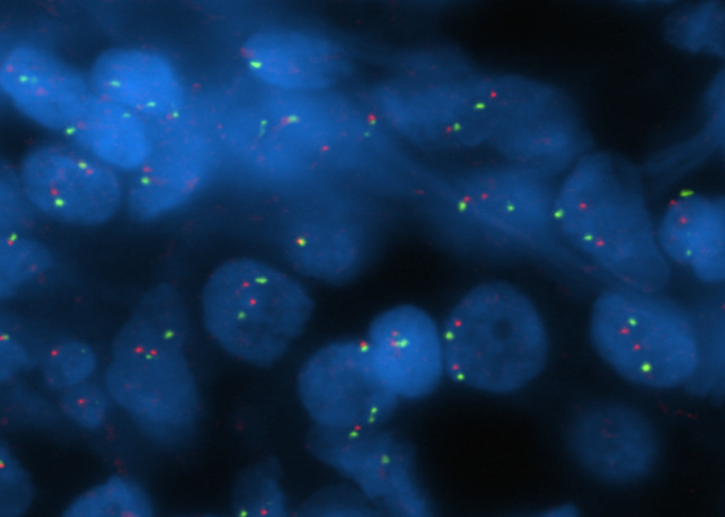


**a**


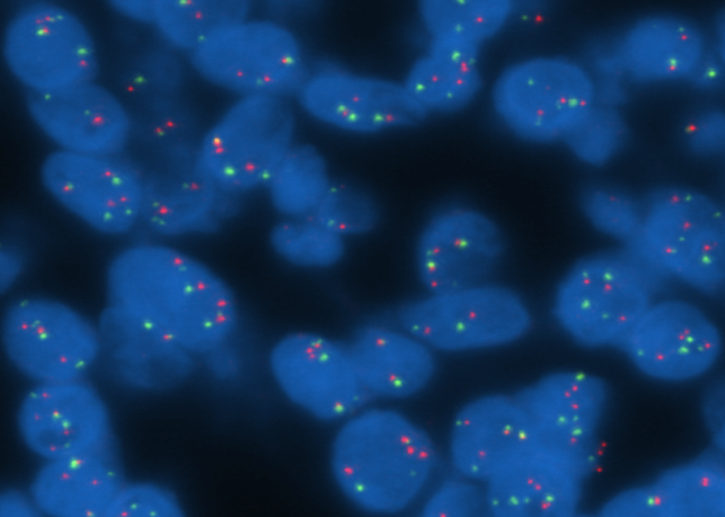


**b**

**
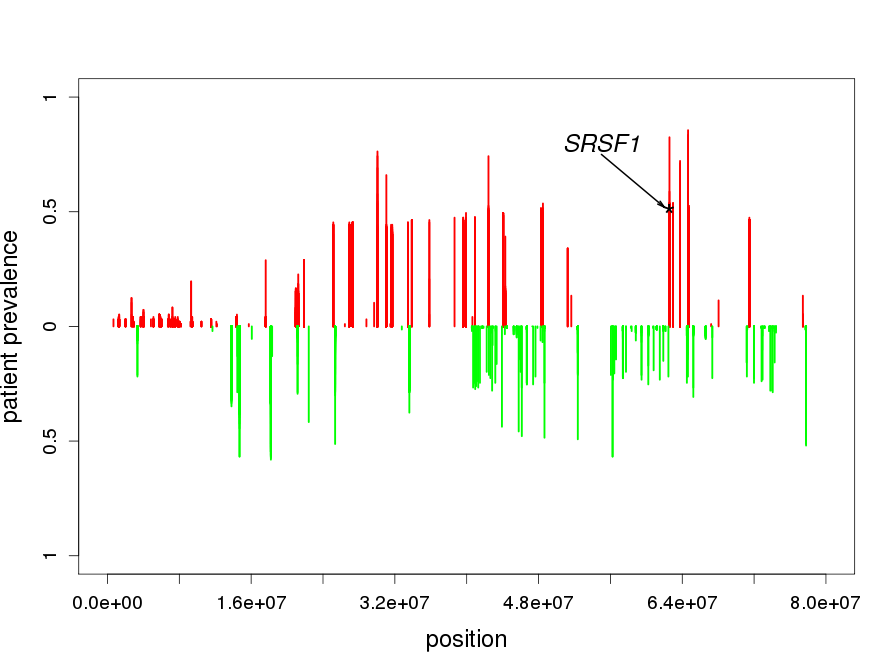
**

**c**

Supplement: S2 Fig — Representative images show a) SRSF1 normal and b) SRSF1 copy number gain. Red signals represent SRSF1 gene and green signals represent of CEP17; c) SRSF1 CNV SCLC patient prevalence as well as other CNV segments across chromosome 17. Red lines indicate CN gains and green lines indicate CN losses. (DOCX) [file pgen.1005895.s002.docx]

**
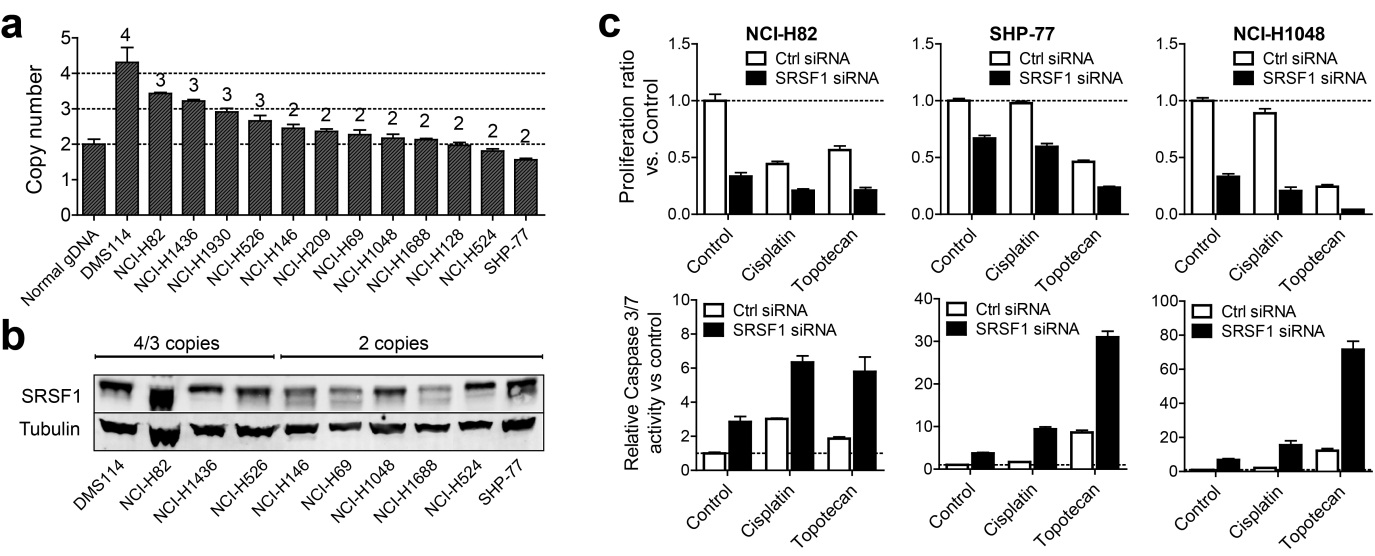
**

Supplement: S4 Fig — (a): TaqMan assays of SRSF1 DNA CNs in 13 SCLC cell lines. (b): Western blots of SRSF1 show protein expression levels in SCLC cell lines. (c):NCI-H82, SHP-77 and NCI-H1048 were transfected with non-targeting control or SRSF1-directed siRNAs for 48 hrs, then treated with cisplatin (2.5ug/ml) or topotecan (2.5ug/ml) for 24 hrs. Cell growth and Caspase-3/7 activities were assessed and normalized against ctrl siRNA-transfected cells as 100% control. (DOCX) [file pgen.1005895.s004.docx]

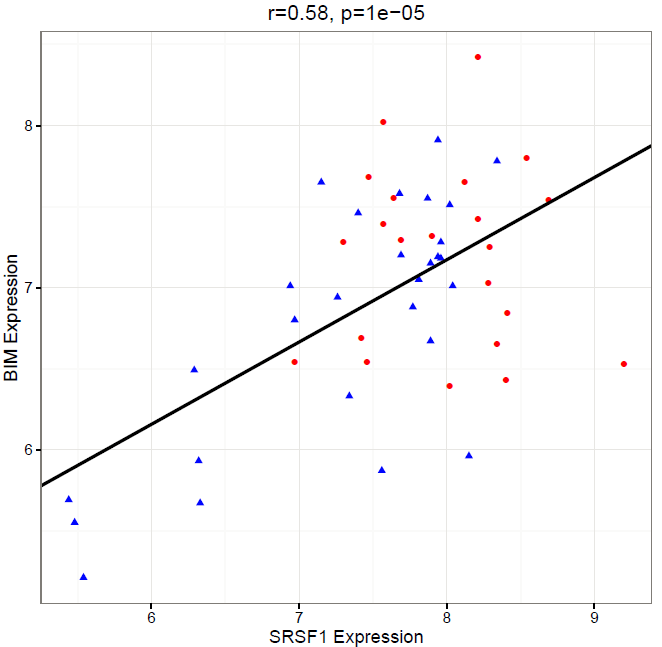

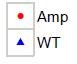


CN Gain / Amp

CN Neutral

Supplement: S5 Fig — The correlation between SRSF1 and BIM gene expression is significant, which likely confirms SRSF1 over expression promotes alternative splicing of BIM. (DOCX) [file pgen.1005895.s005.docx]

**
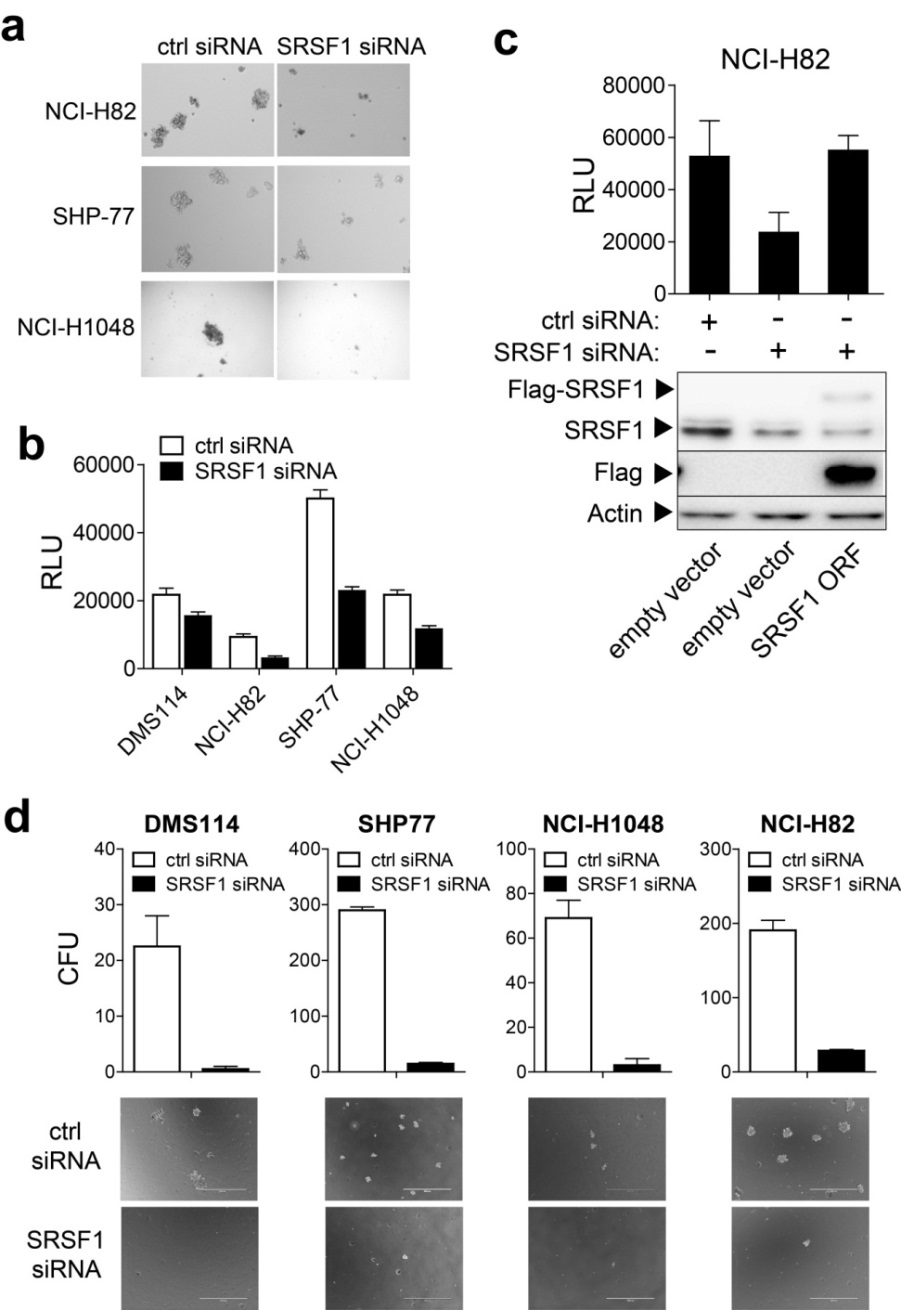
**

Supplement: S6 Fig — (a): Phase-contrast images of the sphere formation under each condition were captured. (b): viable cell mass quantitated by CTG assay. (c): Reconstitution of SRSF1 expression using a siRNA-resistant Flag-tagged SRSF1 expression construct was carried out in SRSF1 siRNA transfected NCI-H82 cells. Impact on sphere growth rate was assessed by CTG assay, and successful SRSF1 protein re-expression was confirmed by WB using either anti-SRSF1 antibody or anti-Flag antibody. (d): Clonogenic assays of DMS-114, NCI-82, SHP-77 and NIH-H1049. Cells were transfected with siRNAs for 48 hrs and then seeded in the methylcellulose medium for 7~14 days, colonies with more than 40 cells per colony were counted. (DOCX) [file pgen.1005895.s006.docx]

**
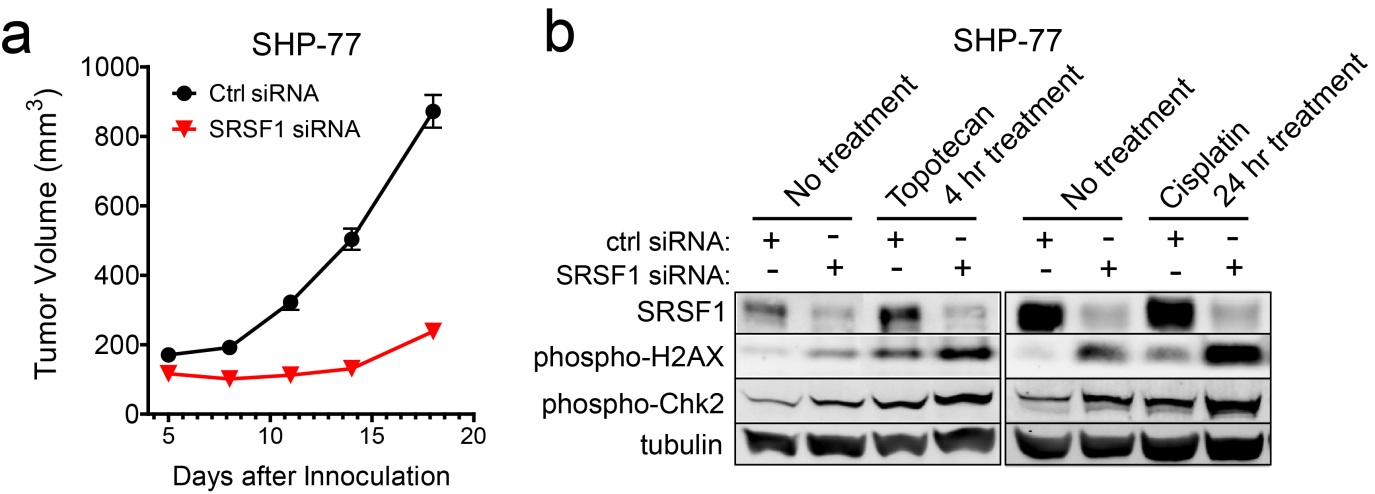
**

Supplement: S7 Fig — (a)SHP-77 cells transfected with non-targeting control siRNA or SRSF1 siRNA were implanted into immunocompromised mice and tumor formation rates were monitored and measured. (b): SHP-77 cells were transfected with control or SRSF1 siRNA and then treated with topotecan or Cisplatin for the indicated times. SRSF1, phosphor-H2AX and phosphor-Chk2 were probed with their corresponding antibodies. (DOCX) [file pgen.1005895.s007.docx]

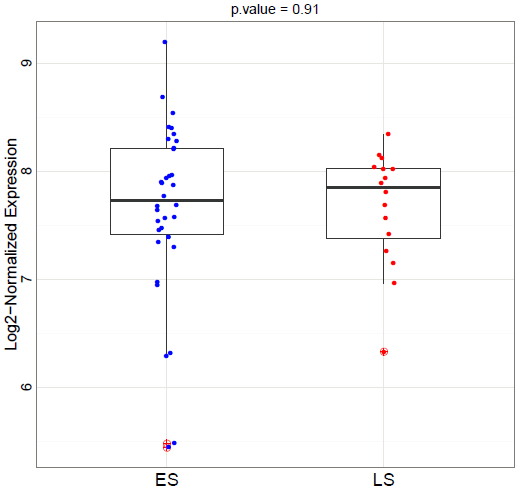

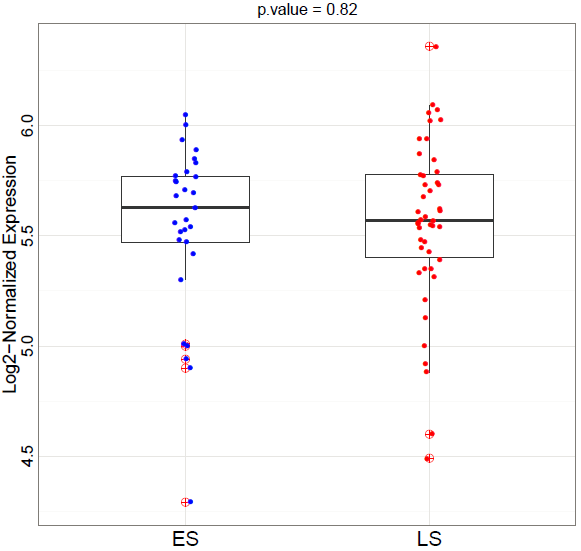


Chinese SCLC

Late Stage

Early Stage

Early Stage

Late Stage

Supplement: S8 Fig — P-value is calculated using Welch’s modified t-test. (DOCX) [file pgen.1005895.s008.docx]

SCLC Cell Line


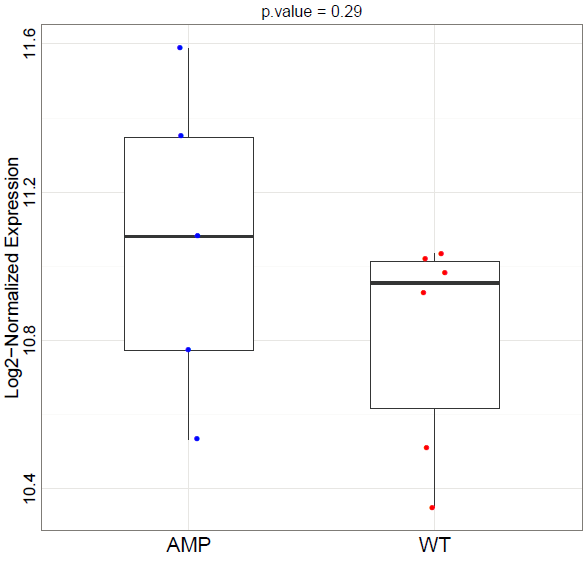

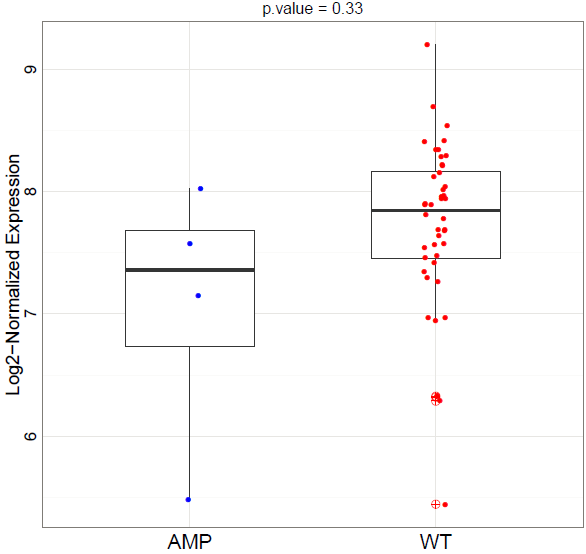


Chinese SCLC

CN Gain / Amp

CN Neutral

CN Gain / Amp

CN Neutral

Supplement: S9 Fig — SCLC cell lines (left figure, N = 11) and our Chinese SCLC study (right figure, N = 49 patients with matched expression and CNV samples). P-value is estimated using standard t-test. (DOCX) [file pgen.1005895.s009.docx]
