# Supplementary material for: Genomic Landscape Survey Identifies SRSF1 as a Key Oncodriver in Small Cell Lung Cancer
Source: PLoS Genet. 2016 Apr 19;12(4):e1005895. doi: 10.1371/journal.pgen.1005895 (PMC4836692; doi:10.1371/journal.pgen.1005895)
Supplement: S3 Fig — Plots were generated in OncoLand (OmicSoft Corp; Cary, NC). (DOCX) [file pgen.1005895.s003.docx]

BRCA

KIRP

SARC

SKCM

UCEC
