# Supplementary material for: Genomic Landscape Survey Identifies SRSF1 as a Key Oncodriver in Small Cell Lung Cancer
Source: PLoS Genet. 2016 Apr 19;12(4):e1005895. doi: 10.1371/journal.pgen.1005895 (PMC4836692; doi:10.1371/journal.pgen.1005895)
Supplement: Supplementary file 3 [file pgen.1005895.s010.docx]

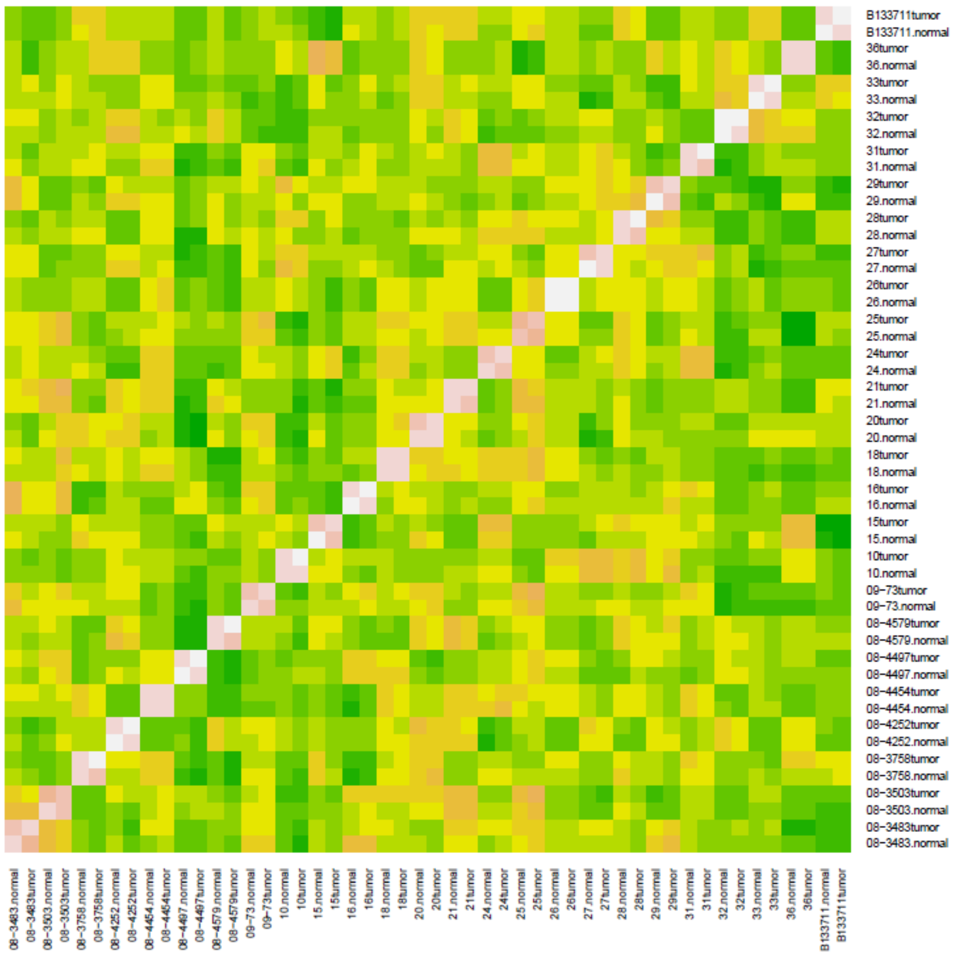
B133711tumor
B133711.normal
36tumor
36.normal
33tumor
33.normal
32tumor
32.normal
31tumor
31.normal
29tumor
29.normal
28tumor
28.normal
27tumor
27.normal
26tumor
26.normal
25tumor
25.normal
24tumor
24.normal
21tumor
21.normal
20tumor
20.normal
18tumor
18.normal
16tumor
16.normal
15tumor
15.normal
10tumor
10.normal
09-73tumor
09-73.normal
08-4579tumor
08-4579.normal
08-4497tumor
08-4497.normal
08-4454tumor
08-4454.normal
08-4252tumor
08-4252.normal
08-3758tumor
08-3758.normal
08-3503tumor
08-3503.normal
08-3483tumor
08-3483.normal
08-3483.normal
08-3483tumor
08-3503.normal
08-3503tumor
08-3758.normal
08-3758tumor
08-4252.normal
08-4252tumor
08-4454.normal
08-4454tumor
08-4497.normal
08-4497tumor
08-4579.normal
08-4579tumor
09-73.normal
09-73tumor
10.normal
10tumor
15.normal
15tumor
16.normal
16tumor
18.normal
18tumor
20.normal
20tumor
21.normal
21tumor
24.normal
24tumor
25.normal
25tumor
26.normal
26tumor
27.normal
27tumor
28.normal
28tumor
29.normal
29tumor
31.normal
31tumor
32.normal
32tumor
33.normal
33tumor
36.normal
36tumor
B133711.normal
B133711tumor
