# Supplementary material for: Genomic Landscape Survey Identifies SRSF1 as a Key Oncodriver in Small Cell Lung Cancer
Source: PLoS Genet. 2016 Apr 19;12(4):e1005895. doi: 10.1371/journal.pgen.1005895 (PMC4836692; doi:10.1371/journal.pgen.1005895)
Supplement: Supplementary file 4 [file pgen.1005895.s011.docx]

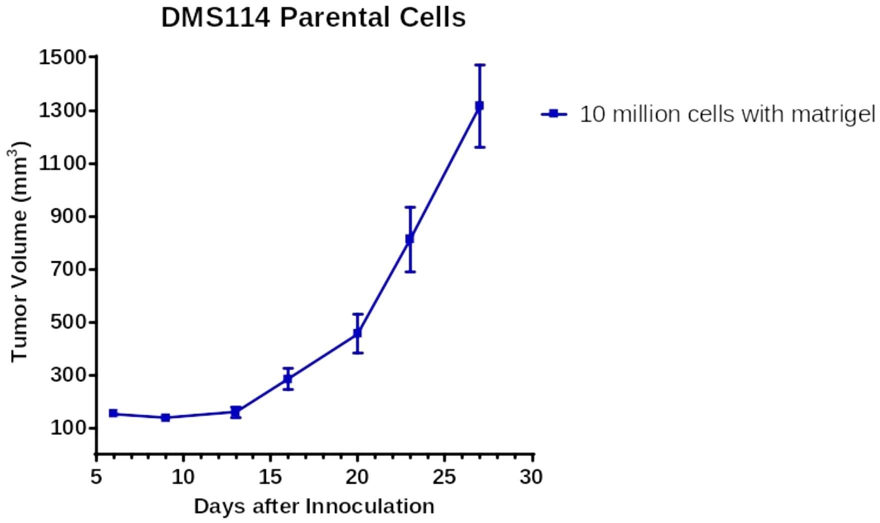

DMS114 Parental Cells
Tumor Volume (mm3)
1500
1300
1100
900
700
500
300
100
5
10
15
20
25
30
Days after Innoculation
10 million cells with matrigel

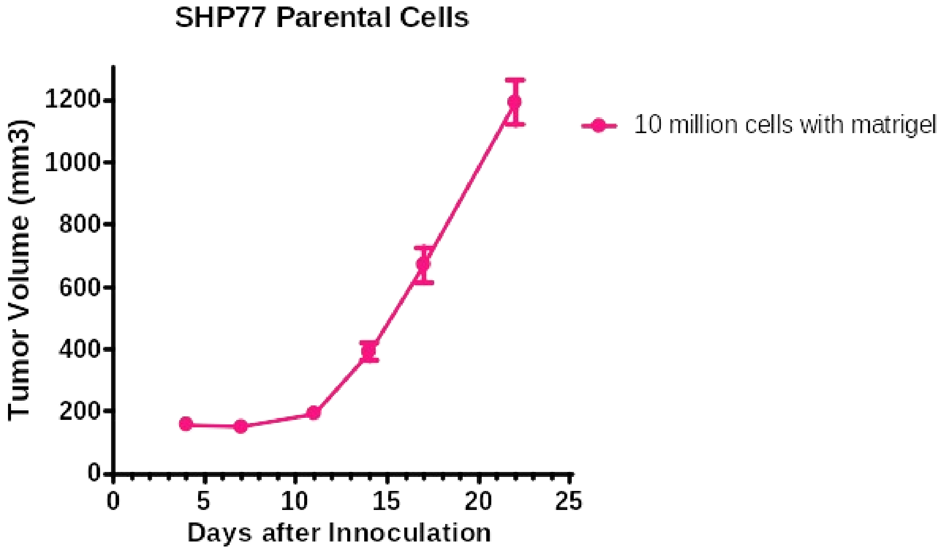

SHP77 Parental Cells
Tumor Volume (mm3)
1200
1000
800
600
400
200
0
0
5
10
15
20
25
Days after Innoculation
10 million cells with matrigel
